# The optimal time to inject bone mesenchymal stem cells for fracture healing in a murine model

**DOI:** 10.1186/s13287-018-1034-7

**Published:** 2018-10-25

**Authors:** Xin Wang, Cheng Wang, Wenlong Gou, Xiaolong Xu, Yu Wang, Aiyuan Wang, Wenjing Xu, Quanyi Guo, Shuyun Liu, Qiang Lu, Haoye Meng, Mei Yuan, Jiang Peng, Shibi Lu

**Affiliations:** 1 Institute of Orthopedics, Chinese PLA General Hospital, Beijing, China; 2 Department of Orthopedics, Peking University Third Hospital, Beijing, China; 3 Department of Orthopaedics, Daping Hospital, The Third Military Medical University, Chongqing, China; 4 Department of Orthopaedics, Xijing Hospital, The Fourth Military Medical University, Xi’an, China

**Keywords:** Homing, Mesenchymal stem cells, Migration, Stromal cell-derived factor 1, Fracture healing

## Abstract

**Background:**

Bone marrow is an important source of stem cells, which can promote bone fracture healing.

**Methods:**

We investigated the optimal time to inject bone marrow mesenchymal stem cells (BMSCs) in a C57 murine unilateral, transverse, femur fracture model. BMSCs transfected with red fluorescent protein (RFP-BMSCs) were injected via the tail vein on day 1, 7, or 14 post-fracture. AMD3100 (inhibitor of stromal cell-derived factor 1 [SDF-1]) was also injected before RFP-BMSCs in one group for comparison; a control group received saline injections. RFP-BMSC migration and fracture healing were evaluated by in vivo fluorescence assay. Micro-CT was performed and mechanical testing and histological analysis. Chemokine levels were evaluated by quantitative real-time PCR and western blotting.

**Results:**

Following injection on day 7 post-fracture, RFP-BMSCs more frequently homed to the fracture site and remained for a longer duration. Bone volume and bone mineral density were increased when BMSCs were injected on day 7 post-fracture (*P* < 0.05). The mechanical properties of fractured femurs were improved following day-7 BMSC injection. Histology confirmed that BMSC injection improved the formation of new bones.

**Conclusions:**

Chemokines that induce BMSC migration were highly expressed, and protein levels of osteogenesis-related factors were increased. Seven days after fracture may be the optimal time for injection of BMSCs to promote fracture healing. Additionally, the SDF-1/CXCR4 pathway may play an important role in fracture healing following BMSC injection.

## Background

Delayed bone union can lead to non-union and severe dysfunction and is difficult to treat [1]. Mesenchymal stem cells (MSCs) are nonhematopoietic stromal stem cells that exhibit multipotent differentiation into chondrocytes and osteocytes [2]. Homing of MSCs may play an important role in the repair of bone fractures. Bone marrow is an important source of stem cells. Many studies have investigated the use of bone-marrow MSCs (BMSCs) therapy to promote fracture healing. Weaver AS, et al. [3] suggested that the relationship between introduction of exogenous MSCs and fracture healing progression may also depend on timing. Chiu LH, et al. [4] elucidated the pivotal role of type II collagen in BMSC osteogenesis and its potential application to bone healing. In these reports, BMSCs were injected at the time of surgery and whether immediate injection is the optimum time for fracture treatment is unknown.

Homing of BMSCs for fracture healing involves MSC recruitment, migration, differentiation, and hyperplasia. Stromal cell-derived factor 1 (SDF-1) is considered a master regulator of C-X-C chemokine receptor type 4 (CXCR4)-positive MSCs [5]. SDF-1 plays a role in the migration of CXCR4-positive MSCs to the site of injury and regulates repair activity [6–8]. Whether SDF-1 plays an important role in fracture repair when BMSCs are injected at different time points post-fracture is unknown. Therefore, we aimed to verify the pathway of MSC homing and explore the effect of injection of stem cells at different time points post-fracture on fracture healing. In addition, we examined the mechanisms underlying the differences in fracture healing after injection of BMSCs at different times post-fracture. We hope to find the appropriate timing for injecting an appropriate dose of BMSCs, which will get better effect of promoting the bone fracture healing. It has great economic and social benefits.

## Methods

### Animals

All experiments followed international regulations for the care and use of laboratory animals and were approved by the Ethics Committee of the General Hospital of the Chinese People’s Liberation Army (China, Beijing, Haidian district, Fuxing road, No. 28). All surgeries were performed under general anesthesia, and all efforts were made to minimize suffering. Adult male C57BL/6 mice (8–10 weeks old) were purchased from the Experimental Animal Center of the Military Medical Science Institute (Beijing). Animals were housed in a light- and temperature-controlled environment and had free access to food and water.

### RFP-BMSCs

C57BL/6 mouse mesenchymal stem cells/red fluorescent protein (RFP-BMSCs, Catalog No. MUBMX-01201, Cyagen Bioscience, Guangzhou, China) were cultured in DMEM medium supplemented with 10% fetal bovine serum, penicillin (100 U/ml), and streptomycin (100 mg/ml) at 37 °C, in a humidified atmosphere containing 5% CO_2_. In general, the growth medium was changed every 3 days. Before injection, cells were trypsin-digested, washed in Hank’s balanced salt solution, centrifuged, and diluted in PBS prior to injection. RFP-BMSCs between passages 3 and 4 were used in all experiments.

### Study design

1. We divided 126 C57BL/6 mice into the following three treatment groups: (1) BMSC—received a saline (0.5 ml) tail vein injection 30 min before injection of RFP-BMSCs (1 × 10^6^/0.5 ml), (2) AMD3100—received AMD3100 (0.01 mM/0.5 ml) injection 30 min prior to RFP-BMSC (1 × 10^6^/0.5 ml) injection, and (3) Control—received two saline (0.5 ml) injections 30 min apart. Injections took place on day 1 (*n* = 48), day 7 (*n* = 48), or day 14 (*n* = 30) post-fracture. Fracture specimens were harvested at days 7, 14, 28, and 42 post-fracture and fixed.
2. Calluses from an additional 15 femur-fractured mice not injected with BMSCs were harvested on days 3, 7, 14, 21, and 28 after fracture, and levels of the following MSC migration chemokines were assayed: SDF-1, transforming growth factor β1 (TGF-β1), and monocyte chemoattractant protein 3 (MCP-3).
3. On day 42 post-fracture, callused were harvested from 15 mice in the three treatment groups injected on day 7 post-fracture, and protein levels of the following osteogenesis-related factors were determined by western blot analysis: bone morphogenetic protein 2 (BMP2), TGF-β1, and vascular endothelial growth factor (VEGF).

### Surgery

After general anesthesia was induced, the right leg was sterilized. The midline anterior knee was incised and a needle of 0.5-mm diameter, flattened to prevent obliteration of the endosteal lining, was inserted in a retrograde fashion up the right femur. The needle tip was recessed within the distal femur to prevent interference with knee joint motion. The incision was irrigated and closed. The right femur was fractured in 120 mice using a 200-g weight dropped 20 cm that depressed a blunt guillotine by 1 mm (half of the diameter of the femur) to produce a standardized closed diaphyseal fracture. Marturano JE et al. [9] reported the fracture model and the author proved the success rate was up to 92.5%. X-rays were taken immediately after the fracture. Mice were administered an intramuscular injection of analgesic (0.05 mg/kg buprenorphine per day) for 3 days and allowed unrestricted weight-bearing and cage activity. Animals were euthanized by CO_2_ asphyxiation unless otherwise indicated.

### Specimen harvesting

Mice injected on days 1 and 7 post-fracture were sacrificed using a CO_2_ chamber on days 14 and 42, while mice injected on day 14 were sacrificed at 42 days post-fracture. The intramedullary needle was removed from the right femur of each mouse.

### Fluorescence imaging analysis

Fluorescence imaging involved the use of an LB 983 NC100 Night OWL imaging system (Berthold Technologies, Germany). Because it is difficult to detect fluorescence under deep muscle, the femur was removed, along with the contralateral femur as a control, on days 7, 14, 28, and 42 post-fracture. Samples were imaged immediately at 600-nm emission and 530-nm excitation using the AMD3100 treatment group as a positive control. Fluorescence signal at the region of interest [10], measured as integrated photons/second/cm^2^/steradian, was normalized by dividing the background signal in a similar region of interest on the contralateral intact femur.

### Micro-CT

Bones were thawed at room temperature, and excess tissue was removed. Femurs were vertically aligned on styrofoam to keep the bones stabilized and separated. Specimens were scanned at 20-μm resolution, 2000 s exposure time, 0.5 angle of increment, and 80 kV and 450 μA with a Micro-CT scanner (RS-9 system; MicroView 2.1 software, GE Healthcare, Ontario, Canada). The scan area was manually adjusted to include only the callus and cortical bone immediately adjacent to the callus in 400 slices.

### 3D evaluation

The volume of interest in the micro-CT image data was the callus, a 6-mm-long section of the mid-diaphysis (250 slices). The volume of interest was defined manually by means of an image segmentation process. For 2D tomograms (transverse callus slices) every 10 slices, the outer boundary of the callus and the periosteal surface of the original cortex was manually delineated and linear interpolation was used to define the callus and cortical boundaries in the intervening tomograms. The volume of interest was defined as the region enclosed by these two contours over all serial tomograms. Thus, this procedure excluded the preexisting cortical bone and the medullary canal volume from the analysis (Fig. 2D). The following were quantified for the volume of region of interest: total volume (TV), bone volume (BV), bone volume fraction (BV/TV)(means callus/ total volume), and bone mineral density (BMD).

### Mechanical testing

At day 42, five specimens from each treatment group were randomly selected for three-point-bend biomechanical testing as described previously [11]. The bone was loaded into the electroforce-based system ELF 5100 (Bose Corp., Framingham, MA, https://www.bose-electroforce.com) with a displacement rate of 0.3 mm/min until bone failure was observed. A force-displacement curve was used to calculate the maximum load, maximum radial degrees, elastic radial degrees, and rigidity using the WinTest Control software [12].

### Quantitative RT-PCR

RNA was extracted from fracture calluses on day 3 and weeks 1, 2, 3, and 4 post-fracture and transcribed into cDNA using ReverTrace qPCR RT Master Mix (TOYOBO Life Science). RT-PCR reactions, with SYBR Green Real-time PCR Master Mix-Plus (TOYOBO Life Science), were run in triplicate using a StepOne Real-Time PCR System (Applied Biosystems, Carlsbad, CA, USA). Cycle parameters included an initial incubation at 95 °C for 60 s, followed by 40 cycles of 95 °C for 15 s, 58 °C for 15 s, and 72 °C for 45 s. PCR amplification of cDNA for the chemokines SDF-1, TGF-β1, and MCP-3 used the mouse-specific primer sets in Table 1 (SBS Genetech, Beijing). GAPDH expression was used as a normalization control. The 2^−ΔΔCt^ method was used to evaluate relative expression levels.

**Table 1:** The list of primers used for q-RT PCR

Gene	Forward primer (5′–3′)	Reverse primer (5′–3′)
SDF-1	AAACTGTGCCCTTCAGATTGTT	CGGGGAACTAGTTTTTCCTTTT
TGFβ1	CAAGTGTGGAGCAACATGTGGAA	ATCAGTGGGGGTCAGCAGCC
MCP3	TCTGTGCCTGCTGCTCATAG	CTTTGGAGTTGGGGTTTTCA
GAPDH	TGACATCAGAAGGTGGTGAAG	ATCCTGTTGCTGTAGCCGTATT

### Western blot analysis

Western blotting was performed as described previously [13]. Briefly, calluses were ground in liquid nitrogen and incubated with RIPA lysis buffer (P0013B, Beyotime Institute of Biotechnology, Shanghai) at 4 °C for 30 min, with shocking every 5 min. Samples were then centrifuged for 10 min at 14,000 rpm at 4 °C. Denatured proteins were separated by SDS-PAGE and transferred onto a PVDF membrane, which was blocked with 5% skim milk in 1× Tris-buffered saline Tween-20 (TBST, Applygen Technologies, Beijing). The membrane was then incubated with a primary antibody (anti-BMP2, anti-TGF-β1, anti-VEGF, or anti-β-actin (all from Abcam, MA, USA)) followed by a horseradish peroxidase-conjugated goat anti-mouse IgG secondary antibody (Beyotime Institute of Biotechnology, Shanghai). The membrane was then exposed to Kodak Biomax-Ml film (Eastman Kodak Co., USA). Signals were quantified by densitometry.

### Histological evaluation

Samples from the three treatment groups injected on day 7 were evaluated histologically on days 14 and 42 post-fracture. For histological assessments, bones were fixed in 10% paraformaldehyde. Specimens were completely decalcified in 14% *w*/*v* ethylenediaminetetraacetic acid (EDTA) changed three times per week for 4 weeks [14]. After decalcification, the demineralized femurs underwent paraffin embedding and serial sectioning at 5-μm intervals. Routine hematoxylin and eosin (H&E) and Masson’s trichrome staining was performed for histological analysis. Images were obtained using an Olympus BX51 microscope equipped with a DP71 camera (Tokyo, http://cn.olympus.com/).

### Statistical analysis

Data are expressed as means ± SD. Statistical analysis was conducted using one-way ANOVA and a least-significant-difference test using the SPSS 13.0 software (SPSS Inc., Chicago, IL). *P* < 0.05 was considered to indicate statistical significance.

## Results

### The presence of CXCR4 is essential for MSC homing to the fracture site

**Fig. 1:**
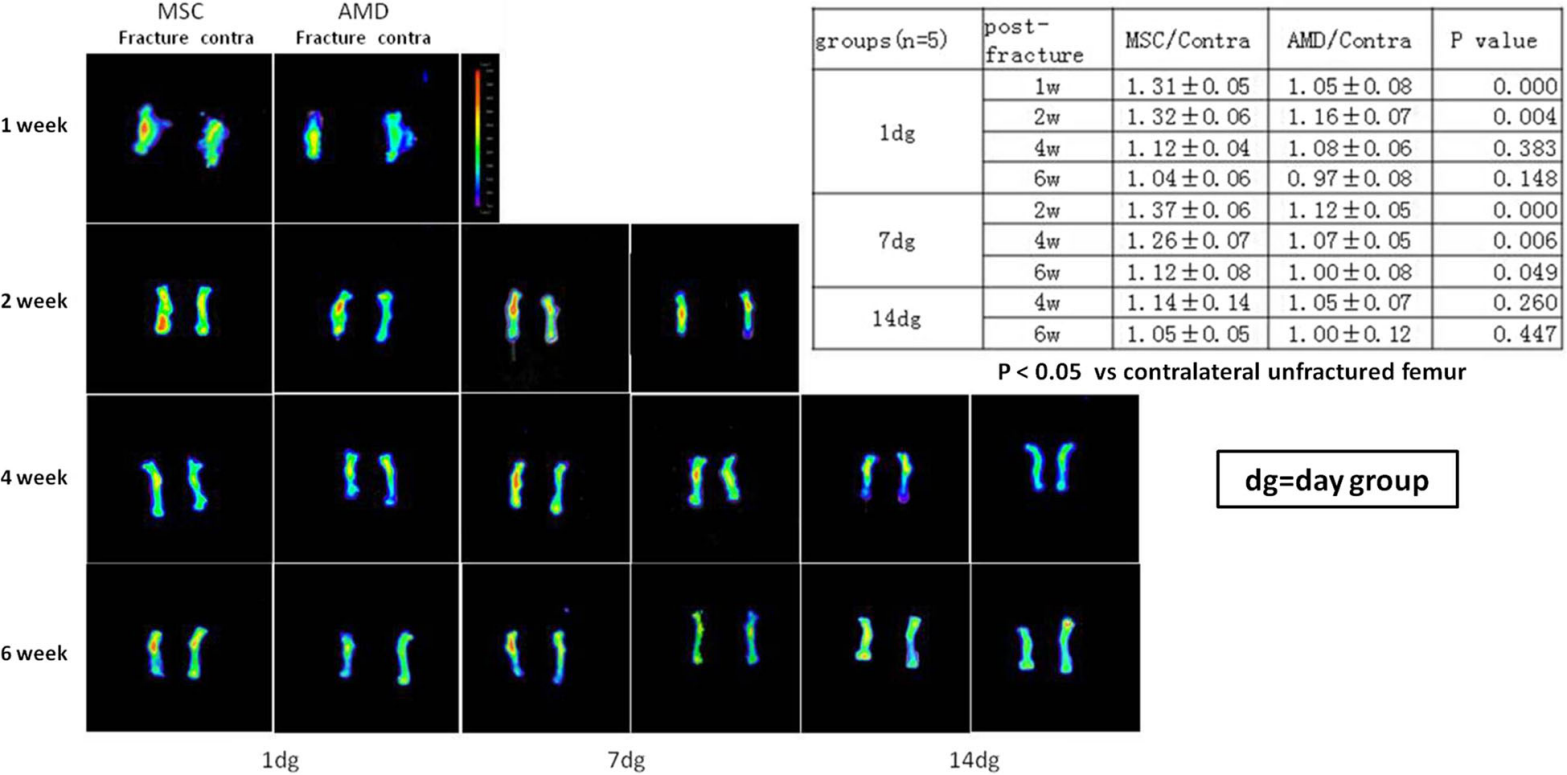
Representative photographs of fluorescence imaging and semiquantitative analysis. **a** Fluorescence was examined at days 7, 14, 28, and 42 after femur fracture in mice, transplantation of bone marrow mesenchymal stem cells (BMSCs) (left panel) or AMD3100 (injected half an hour before BMSC injection, right panel) at 1 day (left column), 7 days (middle column), and 14 days later (right column) after fracture. The fractured and contralateral unfractured femurs are shown in every photo. Graded color bar indicates fluorescence signal intensity expressed as photons/seconds/cm^2^/steradian. **b** Semiquantitative analysis of fluorescence signals. Signal at the fracture site region of interest [7] was normalized to the background signal found in a similar region of interest in the contralateral unfractured femur. (*n* = 6 mice). *P* < 0.05 was considered to indicate statistical significance .(dg = day group)

To assess MSC homing at the femur fracture site, we injected RFP-transfected BMSCs (RFP-BMSCs) into mice on days 1, 7, and 14 post-fracture. In fluorescence imaging analysis, following a day-1 injection, RFP signal intensity was greater on days 7 and 14 when RFP-BMSCs were injected in the absence of AMD3100, although signal intensity was similar in both groups on day 28. A similar pattern of RFP signal intensity was seen after BMSC injection on day 14, although the overall signal intensity was higher following day 7 injection between with and without AMD3100(Fig. 1). At day 42 post-fracture, signal intensity did not differ between BMSC injections on days 1, 7, and 14. Therefore, a higher RFP signal intensity was maintained following RFP-BMSC injection on day 7 post-fracture (*P* < 0.05).

### MSC injection on day 7 post-fracture accelerates and improves fracture repair

**Fig. 2:**
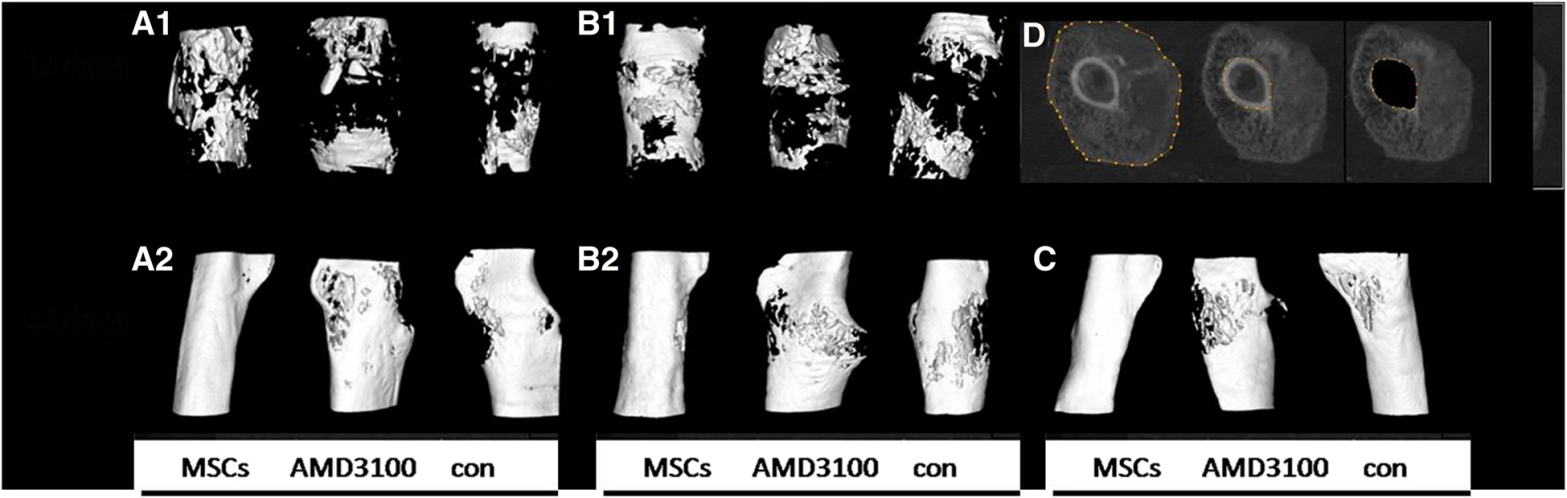
New callus structure at days 14 and 42 after fracture as assessed by micro-CT. (A1, B1) Representative micro-CT 3D reconstructed images of newborn callus at day 14 after fracture (original bone removed). (A2, B2, C) Representative micro-CT 3D reconstructed images at day 42 after fracture. Callus with BMSCs, AMD3100, and BMSCs or saline injected at day 1 (A1, A2), 7 (B1, B2), and 14 (C) post-fracture. (D) The region of interest (highlighted in green) is the newborn callus volume excluding the original cortical bones and the medullary canal volume

**Fig. 3:**
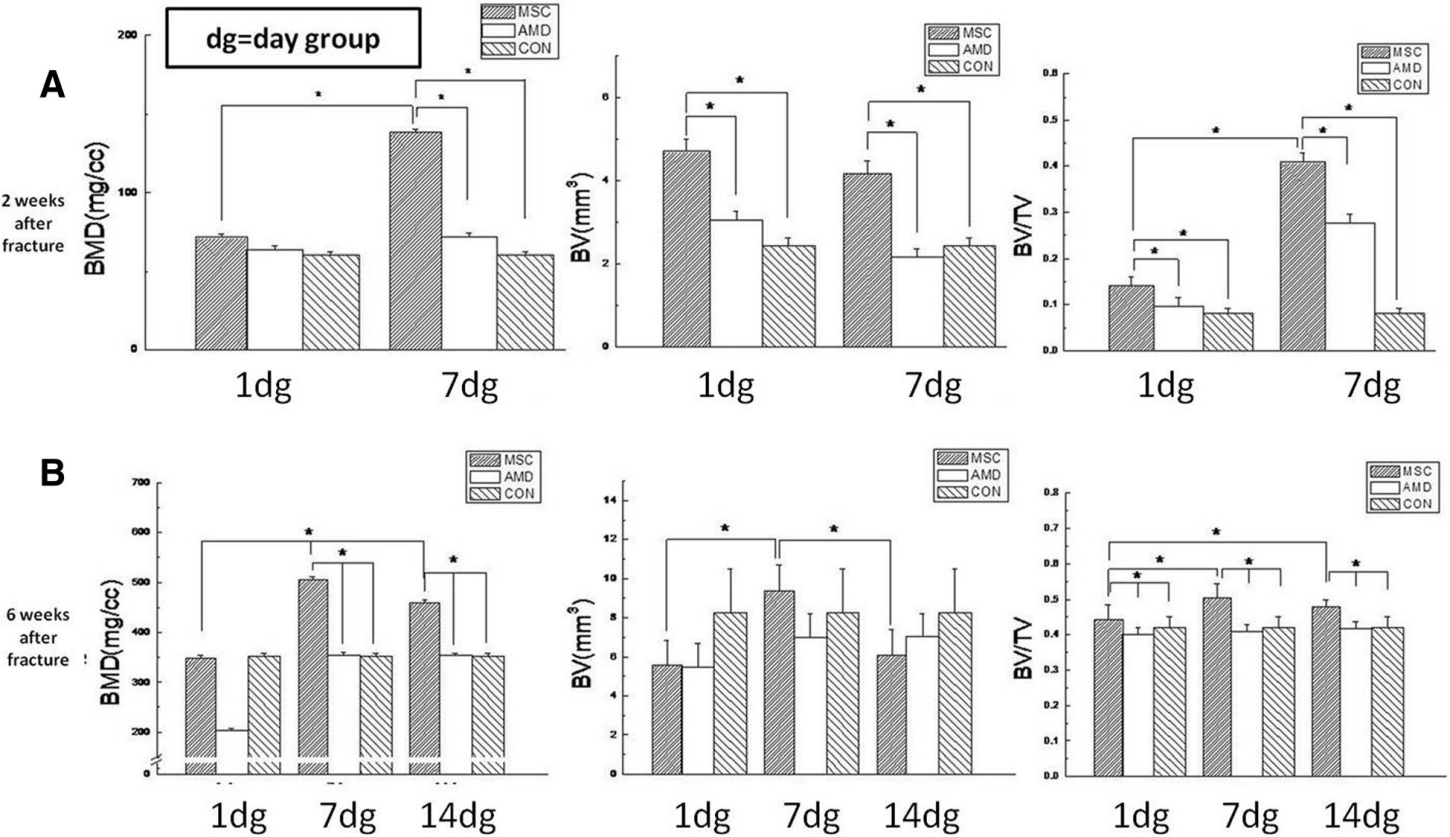
Quantitative analysis of bone mineral density (BMD), bone volume (BV), and bone volume fraction (BV/TV) as determined by micro-CT. **a** Day 14 post-fracture with BMSCs, AMD3100, and BMSCs or saline injected on day 1 and 7. **b** Day 42 post-fracture with BMSCs, AMD3100, and BMSCs or saline injected on days 1, 7, and 14 (*P < 0.05) (*n* = 6 mice). Data are mean ± SD

Three-dimensional reconstructions of entire calluses showed remarkable differences in the size and morphological features of calluses in mice that received BMSCs at different time points. At day 14 post-fracture, callus size, excluding the original bone and marrow (Fig. 2D), was increased following BMSC injection on days 1 and 7 compared to the AMD3100 or control treatment groups (*P* < 0.05) (Fig. 2 A1, B1). At day 42 post-fracture, calluses from mice treated with BMSC injections on days 1, 7, and 14 were being shaped while calluses from mice in the AMD3100 or control treatment groups were in the molding stage (Fig. 2 A2, B2, C). BMD, BV, and bone volume fraction (means callus/total volume) were greater with BMSC injection than in the control treatment (*P* < 0.05) (Fig. 3). BMD and bone volume fraction 14 days post-fracture were higher following BMSC injection on day 7 than on day 1 post-fracture (*P* < 0.05) (Fig. 3a). At day 42, BMD and BV were greater following BMSC injection on day 7 than on day 1 or 14 post-fracture (*P* < 0.05) (Fig. 3b). Therefore, BMSC injection at day 7 post-fracture accelerates the repair process and improves the material properties of the callus by providing more callus bridges between the bone ends than does BMSC injection at day 1 or 14.

### BMSCs improve the biomechanical properties of the fracture callus

**Fig. 4:**
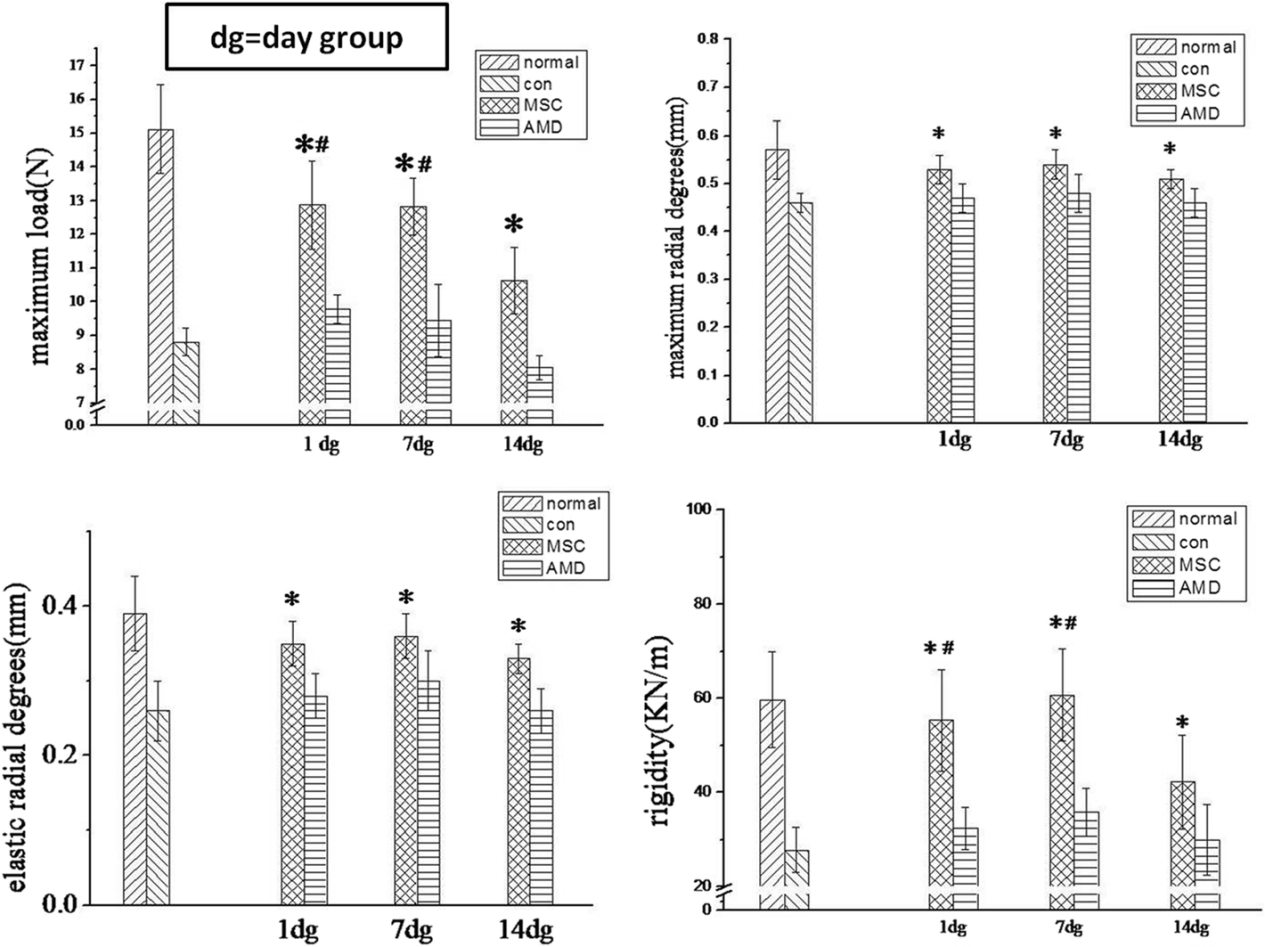
BMSCs improve the biomechanical properties of the fracture callus at 42 days after injection. Quantitative measurements of maximum load, maximum radial degrees (mm), elastic radial degree, and rigidity as determined by three-point-bend biomechanical testing. (*P < 0.05 compared with AMD3100 and control; #*P* < 0.05 compared with BMSC injection at day 14) (*n* = 6). Data are mean ± SD

A key feature of bone healing is tissue regeneration, which provides sufficient strength and functional recovery. We performed three-point-bend biomechanical testing to investigate the material properties of calluses. Calluses harvested 42 days post-fracture from mice injected with BMSCs on days 1, 7, and 14 post-fracture, as well as calluses from the AMD3100 and control treatment groups, were subjected to a gradual increase in distraction force until they broke. Calluses from mice that received BMSC injections demonstrated increased maximum load, maximum radial degrees, elastic radial degrees, and rigidity compared to calluses from mice in the AMD3100 and control treatment groups (Fig. 4). In BMSC-injected mice, the maximum load and rigidity were improved following injections on days 1 and 7 compared to those on day 14 (*P* < 0.05). Maximum and elastic radial degrees did not differ between the injection time points. Therefore, BMSC injection on day 1 or 7 post-fracture results in favorable mechanical properties.

### High-level expression of chemokines associated with BMSC migration on day 7 post-fracture

**Fig. 5:**
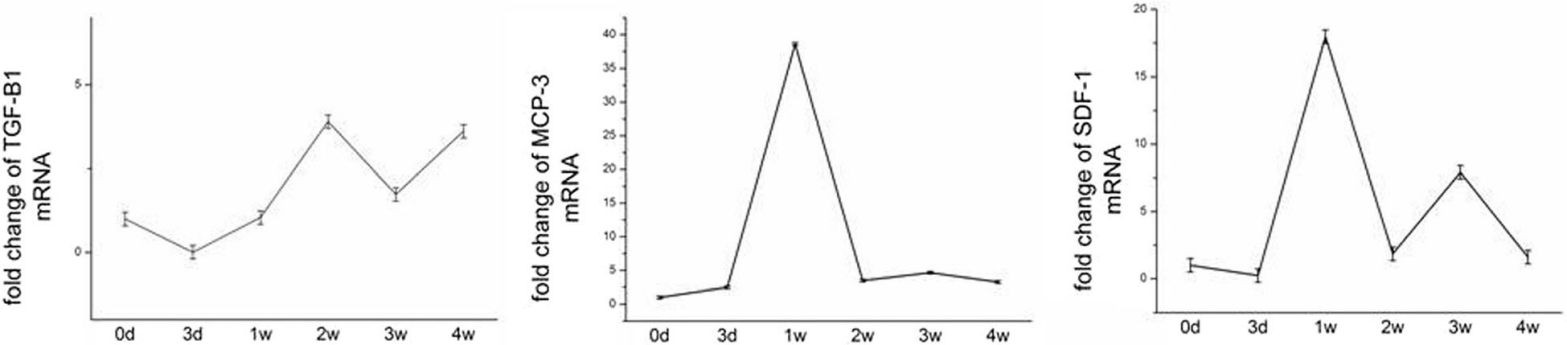
Changes in the gene expression levels of SDF-1, TGF-beta 1, and MCP-3 in callus of the fractured dogs. Stromal cell-derived factor 1 (SDF-1), transforming growth factor β1 (TGF-β1), and monocyte chemoattractant protein 3 (MCP-3) are important chemokines for BMSC migration. Quantitative RT-PCR analysis of mRNA levels was measured by q-RT-PCR from callus after fracture at days 0, 3, 7, 14, and 28 (*P < 0.05 compared with day 3 and 2 weeks) (*n* = 3). Data are mean ± SD

The migration of stem cells is the first step in fracture healing. To determine the effect of the timing of BMSC injection on fracture healing, we investigated chemokine expression in calluses during the fracture healing process. SDF-1, TGF-β1, and MCP-3 are important chemokines for MSC migration. The SDF-1 and MCP-3 mRNA levels were greatest on day 7 after fracture (*P* < 0.05) (Fig. 5). TGF-β1 expression was also increased beginning on day 7 post-fracture.

### BMSCs increase osteoblast differentiation by increasing expression of osteogenesis-related factors

**Fig. 6:**
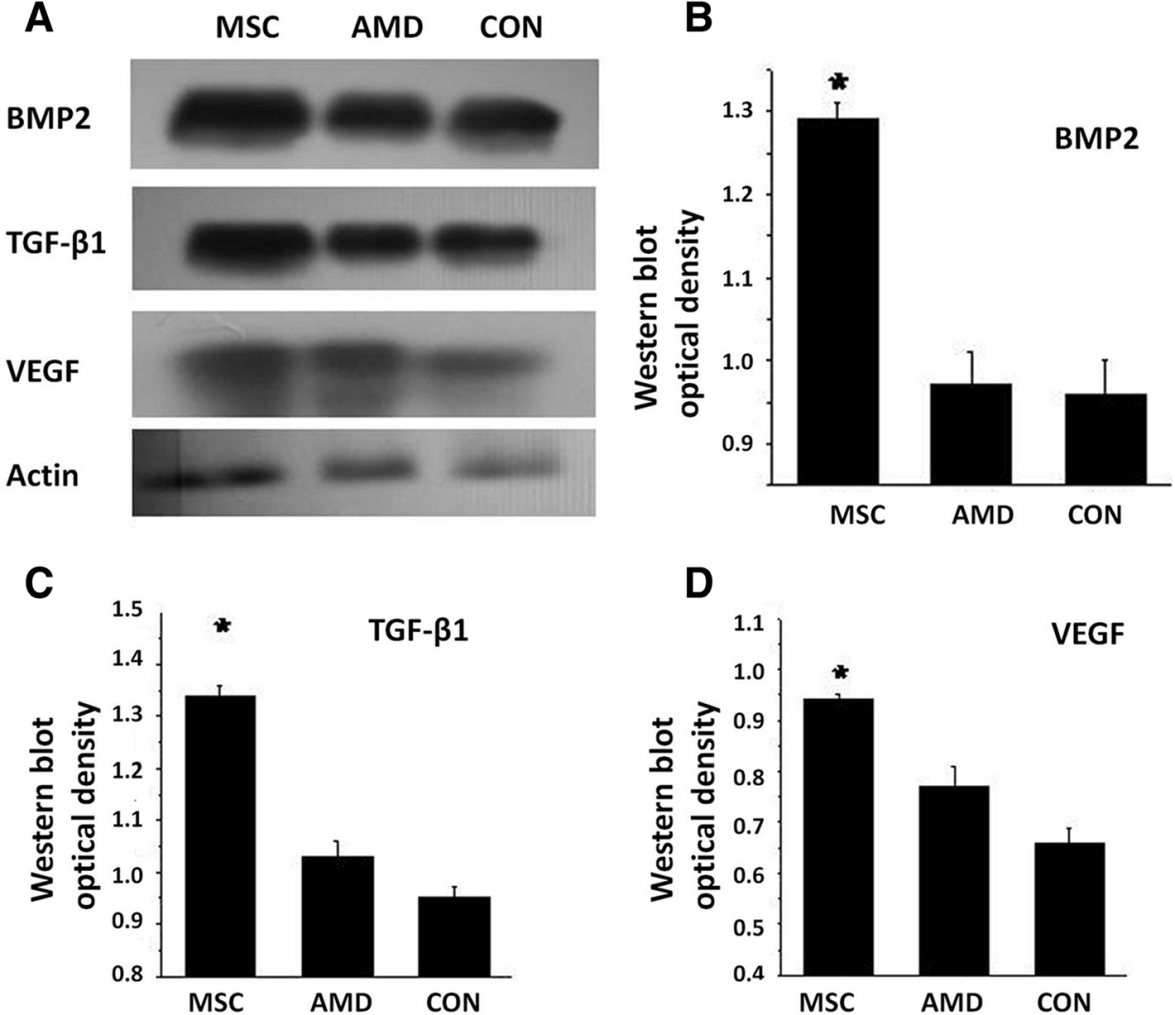
BMSCs increase osteoblast differentiation by increasing the level of the osteogenesis-related factors bone morphogenic protein 2 (BMP2), TGF-β1, and vascular endothelial growth factor (VEGF) at 42 days after fracture. **a** Representative western blot analysis of osteogenesis-related factors compared with AMD3100 and control treatment. **b**–**d** Fold change in protein level of BMP2, TGF-β1, and VEGF. Normalization was to level of actin. *P < 0.05 vs AMD3100 or control group. The gels have been run under the same experimental conditions. Data are mean ± SD from three independent experiments

During the late stage, fracture healing is often accompanied by increased expression of osteogenesis-related factors. Injection of BMSCs improves fracture healing by promoting the expression of osteogenesis-related factors (Fig. 6a), such as BMP2 (Fig. 6b), TGF-β1 (Fig. 6c), and VEGF (Fig. 6d) [15–18]. The sixth week after fracture was the key time point of healing [19–21]. In this time, the expression of the three proteins which promote bone formation in the MSC group was higher than that in the AMD group and control group. Thus we postulated that SDF-1, a master regulator of CXCR4-positive MSC migration to sites of injury and a regulator of repair, might also regulate BMSC survival and proliferation by promoting the expression of osteogenesis-related factors. Elevated SDF-1 levels increased the BMP2, TGF-β1, and VEGF protein levels in calluses on day 42-post fracture, which was abolished by pretreatment with AMD3100, a SDF-1 inhibitor.

### BMSC injection at day 7 post-fracture accelerates callus formation and shortens the fracture-healing process

**Fig. 7:**
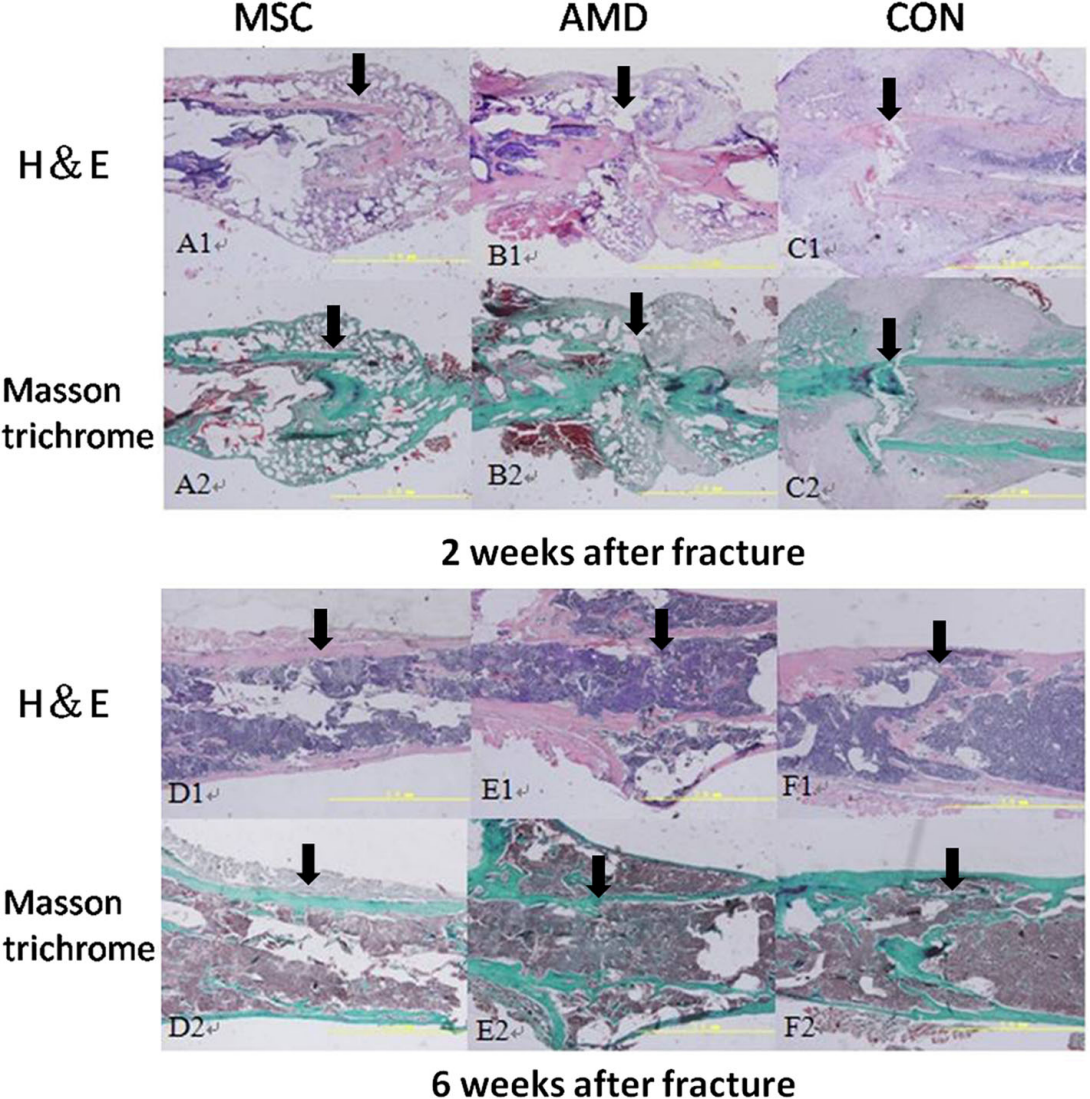
Representative histological samples of fracture calluses at days 14 and 42 after fracture (HE and Masson trichrome stain, magnification, × 40). (Top line) HE stain. (Bottom line) Masson trichrome stain. (Left panel) 14 days after fracture. (Right panel) 42 days after fracture. Callus samples in A1, A2, D1, and D2 were from mice that received BMSC injection at 7 days after fracture. B1, B2, E1, and E2 from mice that received AMD3100 and BMSC injection at 7 days after fracture. C1, C2, F1, and F2 from the control group that received saline injection at 7 days after fracture. More bony calluses were formed in the MSC group than in the AMD group and control group at 14 days after fracture. At 42 days after fracture, bone remodeling had been basically completed in the MSC group, but in the AMD group and control group is still in the shaping process. Black arrows indicate the bone fracture lines

Histology revealed formation of trabecular bony branches near the fracture site, which also shows basic fracture morphology and the situation of bone callus formation. Masson’s trichrome staining was used to visualize calcification of new bone formation from cartilage. Green part mainly contains calcific bone callus. At 14 days post-fracture, samples from BMSC-injected mice produced more bony trabecula and mature cartilage than did samples from the AMD3100 or control group (Fig. 7 A, B, C). At 42 days post-fracture, the marrow cavities of samples from BMSC-injected mice were already unobstructed and bone remodeling had largely been completed (Fig. 7 D, E, F). Injection of AMD3100 blocked this effect. In 42 days after operation, the fracture line in all the three groups disappeared. However, more composition of cartilage and fibrous tissue were in the AMD and control groups.

## Discussion

A previous study demonstrated that 90% of BMSCs undergo apoptosis in the first 3 days after injection; most become trapped in the lungs [22] because the pulmonary capillary diameter is smaller than the diameter of a stem cell [23]. However, in vivo fluorescence experiments have shown that MSCs can arrive at a fracture site via the lung 3 days after injection [15, 24]. In a chemotactic chamber assay to examine the in vitro chemotactic effect of SDF-1 on mouse BMSCs, stem cells with a 30-μm diameter could pass through an aperture with a diameter of 8 μm [25]. Therefore, the alveolar barrier does not limit BMSC migration. The SDF-1 concentration gradient is the driving force behind stem-cell migration and can be blocked by a CXCR4 inhibitor [8, 25, 26]. In our study, we confirmed the dynamic migration of BMSCs in response to fracture in mice and determined that migration to the injury site was driven by CXCR4 regardless of the timing of post-fracture injection. Use of a CXCR4 blocker significantly reduced BMSC homing to the fracture site. Therefore, the SDF-1–CXCR4 pathway plays an important role in BMSC homing for fracture repair.

Fluorescence imaging revealed that BMSCs injected systemically on day 1, 7, or 14 post-fracture migrated to the fracture site. Thus, BMSC transplantation seems to be a valid noninvasive strategy to increase viable progenitors at a fracture site. Although the red fluorescence intensity will gradually decrease along with the gradual healing of the fracture, the three groups have a different timing. In the 7-day group, the time period of the fluorescence intensity was higher than in the control group (AMD3100), which sustained 6 weeks. However, in the 1- and 14-day groups, it was 4 weeks. A high number of BMSCs were specifically recruited to the fracture site, and BMSCs injected on day 7 post-fracture remained at the site for longer than did cells injected at other time points.

MSCs exert their regenerative properties by contributing to each stage of fracture healing [15]. In the early stage of fracture, MSCs effectively inhibit inflammatory factors such as interleukin 1 (IL-1), IL-6, IL-10, and tumor necrosis factor (TNF), to accelerate the fibrous callus formation stage. In the late stage of fracture, MSCs arrive at the fracture endosteum and accelerate bony callus formation and callus remodeling by promoting the expression of BMP2.

At day 7 post-fracture, the acute inflammation period is nearing completion and BMSCs without distraction to overcome inflammation. BMSCs promote chondrogenesis and osteogenesis through the secretion of bone growth factors such as BMP2 and TGF-β1 which leads to lasting proliferation and improved bone formation. BMSCs have autocrine abilities and also promote the paracrine function of osteogenesis-related factors [27]. In the late stage of fracture, the number of exogenous BMSCs decreases but the expression of osteogenesis-related factors increases significantly which suggests that BMSC-induced paracrine effects may be important.

At day 14 post-fracture, mice that received BMSCs on day 7 demonstrated marked differences in terms of the size and morphological features of their new mineralized calluses [24] compared to mice injected on day 1 post-fracture (Fig. 2). The injection of BMSCs on day 7 post-fracture resulted in an increased BMD and bone volume fraction. Therefore, the ratio of new regeneration bone callus to total callus was greater than that after BMSC injection at the other time points.

In our study, BMSC transplantation improved fracture healing by increasing the maximum load, the maximum and elastic radial degree, and rigidity. BMSCs injected at day 7 post-fracture produced tougher calluses than did injections at other time points. The biomechanical observations were consistent with the Micro**-**CT data showing more compact bone calluses following BMSC injection on day 7 than after injection at other time points.

Chemokines play an important role in the homing of BMSCs [28]. One of the best-investigated factors is SDF-1 (a.k.a. pre-B-cell growth-stimulating factor or CXCL12). SDF-1 is considered a master regulator of CXCR4-positive stem and progenitor cells. When MSCs are transported to sites of ischemic injury, this factor modulates the differentiation of MSCs into mature reparative cells [29, 30]. Therefore, the decreased expression of CXCR4 ligand on BMSCs should reduce their proliferation and migration [8]. K. Shinohara et al. [31] reported that SDF-1 and MCP-3 have a synergistic effect, by which they could jointly regulate the homing of MSCs from the systemic circulation to the fracture repair site. This synergistic effect could induce more BMSCs homing to the fracture site and gradually form the precursor cells.

The cells could secrete more factors which promote bone formation and calcification [17, 32]. TGF-β is an effective chemotactic factor for BMSCs [33]. TGF-β1 may play a major role in cartilage formation and endochondral bone formation and therefore could promote BMP2 signaling to enhance osteogenesis and inhibit the activation of osteoclasts by promoting their apoptosis [27]. VEGF and BMP2 exert a considerable synergistic effect on the osteogenic differentiation of MSCs [17, 34]. In our experiment, we detected the factors related to bone formation in the sixth week after operation. The result showed that the MSC group (injecting on seventh day after operation) was apparently higher than the AMD and control groups. It proved that injecting BMSC on the seventh day after fracture could effectively promote fracture healing and all the progress could be interdict by AMD. Chemokine-stimulated BMSC homing leads to the production of osteogenesis-related factors and improved fracture healing. By PCR text, we proved that SDF-1 and MCP-3 reached peak on the seventh day after fracture. TGF-β1 reached peak in the 2 weeks but it also had a high expression on the seventh day. We think that the fracture has the maximum capacity of recruitment of stem cells in this timing. In vivo fluorescence experiment also proved that injecting MSC on the seventh day after fracture could make more stem cells gather to the fracture area. Micro-CT and biomechanical results also proved that it could accelerate the fracture healing process. So, we think that the seventh day is the optimum time point for injecting MSC.

## Conclusion

We conclude that injection of BMSCs can improve fracture healing. BMSCs injected on day 7 post-fracture promoted accelerated fracture healing with improved callus quantity and bone quality. The SDF-1–CXCR4 pathway plays an important role in the fracture healing process. MSCs have many positive effects and may be useful for treatment of delayed fracture healing and bone non-union.

## Abbreviations

BMD: Bone mineral density
BMP2: Bone morphogenetic protein 2
BMSCs: Bone marrow MSCs
BV: Bone volume
BV/TV: Bone volume fraction
CXCR4: C-X-C chemokine receptor type 4
IL-1: Interleukin 1
MCP-3: Monocyte chemoattractant protein 3
RFP-BMSCs: BMSCs transfected with red fluorescent protein
SDF-1: Stromal cell-derived factor 1
TGF-β1: Transforming growth factor β1
TNF: Tumor necrosis factor
TV: Total volume
VEGF: Vascular endothelial growth factor
